# CHALLENGES AND OPPORTUNITIES FOR RECRUITMENT AND RETENTION OF OLDER HISPANICS FOR CLINICAL RESEARCH

**DOI:** 10.1093/geroni/igad104.1309

**Published:** 2023-12-21

**Authors:** Rafael Samper-Ternent

**Affiliations:** UTHealth Houston, Houston, Texas, United States

## Abstract

Older Hispanics represented 9% of the population over 65 years of age in the USA in 2019. That number is expected to increase to 21% by 2060. Representation of older Hispanics in clinical research is not proportional to the projected increase of older Hispanics. I will discuss the lessons learned on recruitment and retention of older Hispanics and their family caregivers from a large multi-site pragmatic clinical trial I am a part of and a small pilot project I am co-leading. The pragmatic trial compares a health-system-based approach and a community-based approach to dementia care. For this pragmatic trial, 2,176 dyads of persons living with dementia and their family caregivers were recruited. More than 20% of participating dyads belong to underrepresented racial and ethnic groups and 8.8% identified as Hispanic. The pilot project tests the cultural adaptation of the Patient Priorities Care (PPC) approach for older Hispanics. For the pilot project, we recruited 5 older Hispanics with multiple chronic conditions (MCC) and 20 Hispanics with MCC and dementia. I will describe the recruitment strategies used in each study, the barriers we faced, and the approaches we think worked best for the recruitment of Hispanics. I will also reflect on the challenges we currently face to conduct pragmatic trials that include older Hispanics and the opportunities for improvement.

